# Choroidal infarction following ophthalmic artery chemotherapy

**DOI:** 10.1186/s40942-018-0119-x

**Published:** 2018-04-30

**Authors:** Kelley J. Bohm, Y. Pierre Gobin, Jasmine H. Francis, Gabrielle McInerney, Anahita Dabo-Trubelja, Paul H. Dalecki, Brian P. Marr, David H. Abramson

**Affiliations:** 1 Ophthalmic Oncology Service, Memorial Sloan Kettering Cancer Center, 1275 York Ave, Room A330, New York, NY 10065 USA; 2 Interventional Neuroradiology, Departments of Radiology and Neurosurgery, Weill Cornell Medical Center/New York Presbyterian Hospital, New York, NY USA; 3 Department of Anesthesiology, Weill Cornell Medical Center/New York Presbyterian Hospital, New York, NY USA; 4 Department of Anesthesiology and Critical Care Medicine, Memorial Sloan Kettering Cancer Center, New York, NY USA

**Keywords:** Choroidal infarction, MTHFR, Nitrous oxide, Ophthalmic artery chemotherapy, Retinoblastoma

## Abstract

**Background:**

Methylenetetrahydrofolate reductase (MTHFR) genetic mutations and intra-procedural inhaled nitrous oxide (N_2_O) independently increase blood levels of homocysteine, a compound associated with thrombosis. Patients with MTHFR mutations who also receive N_2_O during ophthalmic artery chemotherapy (OAC) for retinoblastoma may have a heightened thrombotic risk.

**Case presentations:**

Single-center retrospective review of pediatric patients with advanced retinoblastoma who received OAC and developed choroidal infarcts. Four retinoblastoma patients with advanced intraocular disease (2 males, 2 females: 13–58 months) experienced choroidal infarcts within the one-month period after OAC, in which procedural N_2_O induction was used (duration between 21 and 58 min). All 4 patients had MTHFR (chromosome 1p, position 36.22) genetic abnormalities: one was homozygous for the C677T mutation, one was C677T heterozygous, one was A1298C heterozygous, and one was heterozygous for both C677T and A1298C. In all 4 patients, indirect ophthalmoscopy and fundus photography showed marked disturbance of the retinal pigment epithelium and optical coherence tomography (OCT) confirmed thinning of the choroid. Follow-up time ranged from 15 to 46 months (median 21 months).

**Conclusions:**

Choroidal infarction in eyes treated with OAC developed in children who were both deficient in at least one working allele of the MTHFR gene (heterozygous or homozygous) and received N_2_O induction during OAC.

## Background

**Fig. 1:**
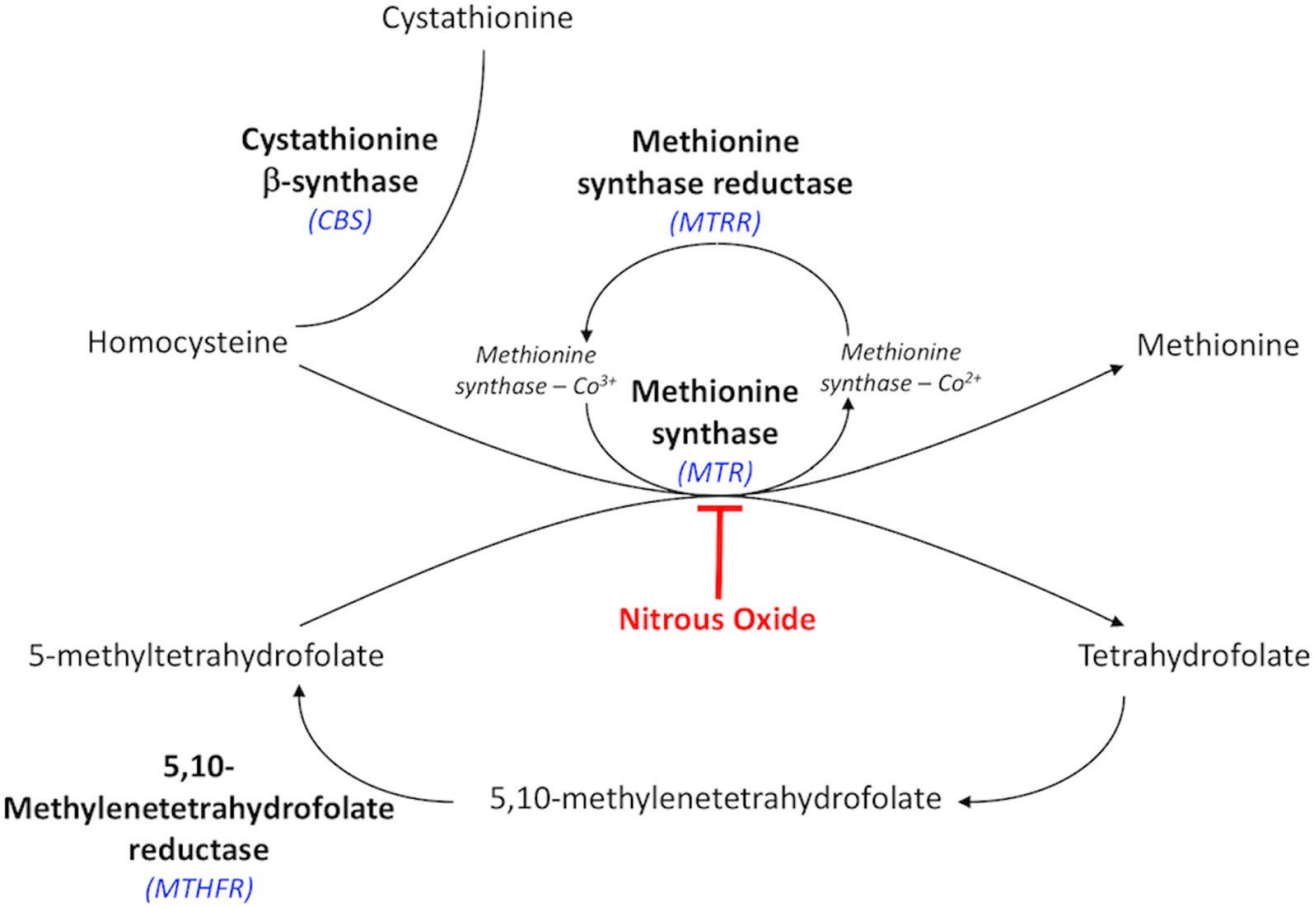
Homocysteine—MTHFR biochemical pathway. Increase of homocysteine can be caused by inhibition of methionine synthase by nitrous oxide and by inhibition of MTHFR by genetic polymorphisms

Choroidal blood flow can be affected by severe hypertension, inflammatory vasculopathies, and thrombophilias (inherited, developed, or iatrogenic). Though choroidal infarction typically entails a lobular and patchy distribution of ischemia, we have seen several cases of choroidal infarction covering many clock hours from the central posterior pole to the periphery after ophthalmic artery chemotherapy (OAC) delivery. In addition to their unique patterns of infarction, these patients had two other curious similarities: methylenetetrahydrofolate reductase (MTHFR) genetic mutations (both heterozygous and homozygous for mutated alleles) and intra-procedural inhaled nitrous oxide—two characteristics known to increase homocysteine in the blood (see biochemical pathway in Fig. 1) [1, 2], thus possibly increasing thrombotic risk [1]. Through this case series, we will describe our experiences and explore the etiology of the patients’ choroidal damage.

## Case presentations

We retrospectively reviewed four retinoblastoma (RB) pediatric patients with MTHFR mutations (of at least one copy of either C677T or A1298C polymorphisms of chromosome 1p at position 36.22). Out of the ten patients our institution’s RB patient cohort who suffered from choroidal infarctions, 7 patients received testing and were positive for MTHFR mutations—4 of whom received OAC and nitrous oxide that corresponded with the timing of infarction. These four patients were diagnosed with choroidal infarctions by imaging at the post-procedural follow-up visits approximately one month after OAC. All patients had been diagnosed with advanced intraocular RB (International Classification Groups D and E) and were monitored regularly at Memorial Sloan Kettering Cancer Center (median length of post-infarct follow-up 21 months, range 15–46 months) and received OAC at New York Presbyterian-Cornell in New York, NY. Prophylactic heparin (dose-adjusted to each patient’s activated clotting time) was administered at the beginning of the procedure and is standard of care for our OAC procedures. Genetic testing for known prothrombotic mutations (in genes for MTHFR, Factor V Leiden, prothrombin) was performed after infarctions were detected. Indirect ophthalmoscopy, RetCam digital photography, optical coherence tomography (OCT) and in some of the patients, fluorescein angiography was used to describe fundus findings. Characteristic findings in choroidal infarction include segmental pallor and granular fundus pigmentation on ophthalmoscopy, thinning of the choroid on OCT, and lack of choroidal perfusion on fluorescein angiography. Below, we will discuss each patient’s characteristics, treatment course, and infarct presentation.

Patient one was a 52-month old male with bilateral RB at the time of infarct diagnosis in his right eye. He was homozygous for the C677T polymorphism, and experienced choroidal ischemia after his seventh dose of OAC (carboplatin, topotecan, and melphalan) to the right eye only. His prior six OAC doses were unremarkable except for vasospasm of the ophthalmic artery during the fifth dose, which limited the ability for simultaneous bilateral chemotherapy infusion. The seventh dose, however, was his first administration of nitrous oxide during the procedure; he received 24 min of nitrous oxide induction anesthesia with a maximum end tidal nitrous oxide (ET N_2_O) of 60.2% (higher ET N_2_O percentage correlates to higher nitrous oxide concentration in the blood). He experienced no intra-procedural complications. One month later, new choroidal ischemia was identified in the right eye, presumed to be due to an infarction.

Patient two was a 13-month old female with bilateral RB when she experienced ischemia of her left choroid. She was heterozygous for the A1298C polymorphism and experienced an infarction after her third cycle of carboplatin OAC to the left eye only, anesthetically induced by nitrous oxide. Two prior OAC infusions were done with sevoflurane induction alone. She received 47 min of nitrous oxide, with 65.6% max ET N_2_O, and the procedure was without acute complications. Follow-up 1 month later revealed infarction of the lateral half of the posterior choroid.

Patient three was a 25-month old male with unilateral RB when he experienced choroidal infarction in the treated eye. His infarction was diagnosed at the one-month follow-up appointment after his second dose of OAC with melphalan and carboplatin. He was heterozygous for two polymorphisms: C677T and A1298C. While he did not receive nitrous oxide during his first OAC session, he did receive 58 min of nitrous oxide with max ET N_2_O 31.9% during this second session of OAC.

Patient four was a 58-month old female when her right eye experienced a choroidal infarction following OAC for her bilateral RB. Her genetic testing showed heterozygosity for the C677T polymorphisim. The infarct occurred after her eighth dose of OAC with carboplatin, topotecan, and melphalan to the right eye (tenth treatment of OAC for this patient, two treatments only to left eye). This was her first time receiving nitrous oxide (duration 21 min, max ET N_2_O 60.5%) during OAC for the right eye. This patient’s choroidal ischemia was identified by RetCam images at her one-month follow-up appointment.

**Fig. 2:**
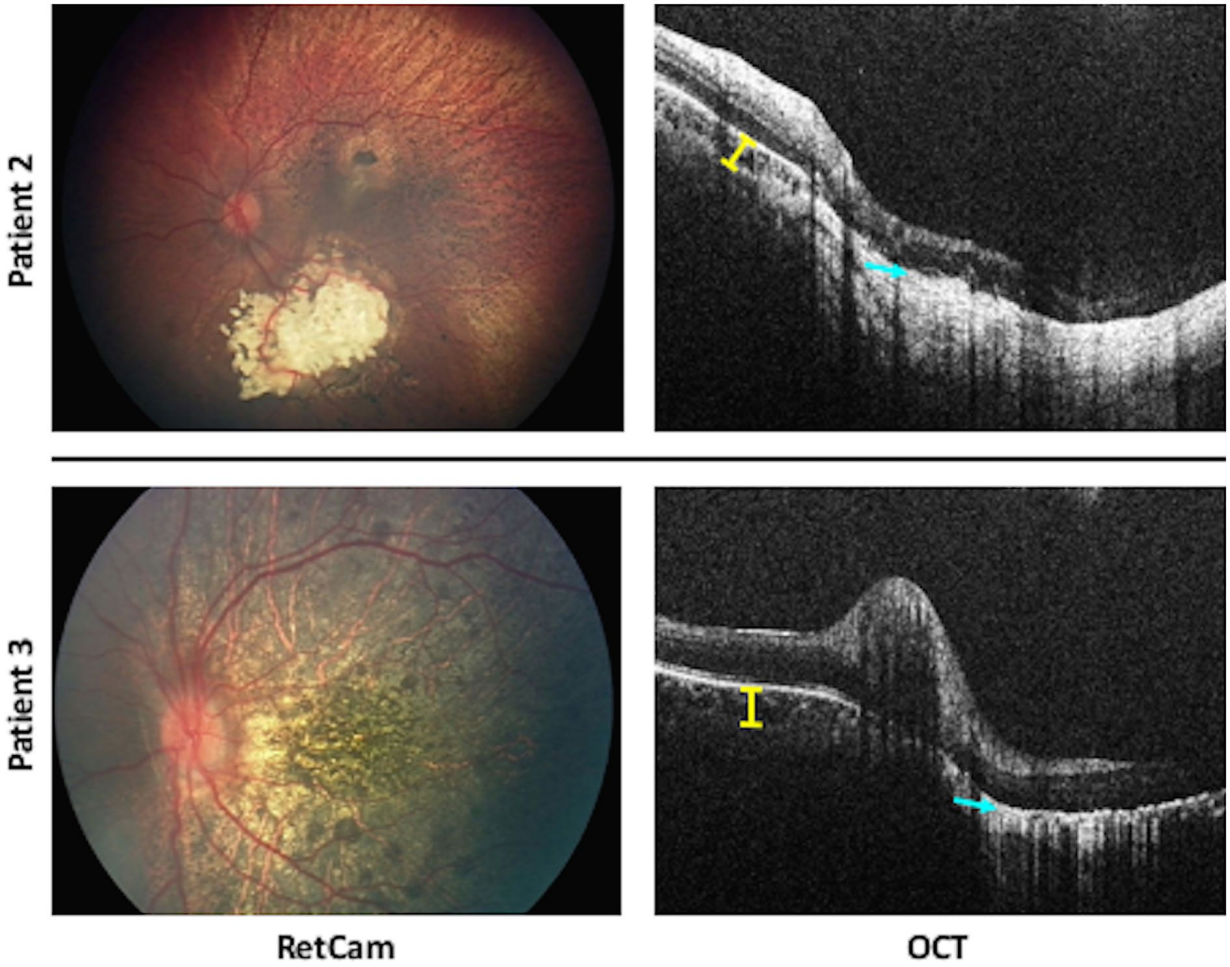
RetCam retinal photographs and OCT choroidal imaging of Patients 2 and 3 one month after OAC with nitrous oxide. Yellow bars indicate choroidal thickness in area not affected by choroidal infarction. Teal blue arrows point to location of thinned choroid in area of infarction

There were no systemic thrombotic events in these patients following OAC. Post-OAC cerebral angiograms performed in each patient (standard of care) showed no evidence of vasospasm in the ipsilateral arteries. In each patient, these angiograms confirmed anterograde flow in the internal carotid artery, anterior cerebral artery, and middle cerebral artery, as well as patent ophthalmic arteries. In all four patients, indirect ophthalmoscopy and fundus photography 1 month after OAC showed marked disturbance of the retinal pigment epithelium and OCT confirmed distinct thinning of the choroid. In all four patients, the choroidal damage covered the lateral 180° from the central pole to the periphery. See Fig. 2 for a representative RetCam fundus photographs and OCT images from patients 2 and 3. Post-infarct brain magnetic resonance images were performed in patients with bilateral RB (1, 2, and 4) to monitor for pinealoblastoma, and no past cerebrovascular events were identified.

## Discussion and conclusions

Ophthalmic artery chemotherapy is an effective oncologic treatment; however, side effects do exist, ranging from temporary lid swelling, strabismus, and loss of eye lashes [3] to less frequent substantial adverse events such as vitreous hemorrhages [4], sectoral choroidal occlusive vasculopathy [5], and choroidal ischemia, as seen in our patients. Post-OAC vascular obstruction and subsequent ischemia has also been attributed to substances such as cotton and synthetic fibers when Melphalan isn’t properly filtered [6], particles from unfiltered melphalan solutions in experimental animals [7], and thromboses [5, 6]. We suspect our patients to have experienced thromboembolic choroidal infarctions based on the timeline and clinical exam and two other key considerations. We have previously reported thrombotic events due to sickle cell trait, prothrombin mutation, and plasminogen activator inhibitor-1 polymorphism [8, 9]. Even though sickle trait, Factor V Leiden, Protein C and S deficiencies, and prothrombin mutations are some of the most common thrombophilic conditions (see Table 1), our patients all exhibited genetic mutations of MTHFR (heterozygous or homozygous) which has an estimated prevalence in the United States of 11% for homozygotes and 40% for heterozygotes [13]. This enzyme mutation causes a moderate increase in circulating homocysteine (though to a lesser extent than its homozygous counterpart), which has shown to cause endothelial dysfunction [2] and a dose-dependent causality association with thromboembolisms [1]. We unfortunately cannot comment on the population with the treatment who did not experience choroidal infarctions because our center does not routinely, prospectively test for MTHFR (and other prothrombotic) mutations. The patients in our report also all received inhaled nitrous oxide during the procedure, a gas that inhibits methionine synthetase activity by 50% during a 2-h procedure and independently causes a dose-dependent increase of homocysteine [2], thereby increasing the risk of thrombosis [14]. A potential synergistic effect of at least one mutated MTHFR allele and nitrous oxide on blood levels of homocysteine in conjunction with the drugs’ intrinsic vascular toxicity may have increased the clotting propensity sufficiently to result in arterial thrombosis and subsequent segmental choroidal infarctions. Homocysteine levels were not obtained at the time of OAC in our patients because of the retrospective nature of this study, however the aforementioned studies enable us to infer homocysteine elevation.

**Table 1:** Prevalence and relative risk of inherited thrombophilia in the general population [9–12]

Coagulation disorder	Prevalence in the general population (%)	Relative risk of venous thromboembolism
Anti-phospholipid syndrome	5–10	5–10
Hyperhomocysteinemia (MTHFR homozygote)	11	2.5
Factor V Leiden (heterozygote)	5	6–8
Prothrombin G20210A mutation (heterozygote)	2.3	2.8
Protein S deficiency	1.3	2.4
Sickle cell trait	1	1.5
Protein C deficiency	0.2	6.5
Antithrombin deficiency	0.02–0.04	17.5

Though many occlusive etiologies can logically explain the choroidal damage in our four patients, the uncharacteristic distribution of the infarct begs the question—was the choroidal damage the result of an infarction at all? Infarctions typically cause smaller, lobular areas of ischemia at affected choriocapillaries, while the infarctions seen in our patients were larger, segmental territories. There were also no systemic ischemic events in these patients (though we previously reported a patient with systemic vascular manifestations [9]), thus a purely microthrombotic event does not convincingly explain these choroidal infarctions. Chemotherapeutic agents (varying combinations of melphalan, topotecan, and carboplatin) have been linked to pH-related toxicity (melphalan and carboplatin are acidic) to the ocular blood vessels, particularly retinal endothelial cell inflammation and leukostasis, following drug administration [7]. Melphalan has shown a direct choroidal toxicity as the result of preferential drug uptake of the retinal pigmented epithelium and poor efflux to the retina [15]. This, however, does not explain why the toxicity only affected one portion of the eye, rather than the entire territory fed by the ophthalmic artery. The patients may also have had pre-existing retinal detachments enabling chemotherapy accumulation and choroidal toxicity (though no serous retinal detachments were visualized on examination) [16], dietary deficiencies (i.e. vitamin B12, a necessary cofactor for the MTHFR pathway), or metabolic derangements.

These alternative explanations highlight important considerations, yet they don’t offer a unifying reason why these patients were affected and not others. We suspect that these four patients suffered visually significant choroidal complications following OAC due to a combination of their genetic predisposition to form blood clots and the nitrous oxide administered during these procedures for induction anesthesia focally in the setting of local melphalan. We hope that our observation on the association between MTHFR abnormalities and exposure to nitrous oxide exposure during OAC for RB will lead centers to prospectively study the incidence of MTHFR abnormalities, homocysteine levels before and during procedures and overall relationship to choroidal infarcts. It would be reasonable to consider withholding nitrous oxide in these children during OAC until additional information can rigorously determine its importance.

## Abbreviations

MTHFR: methylhydrofolate reductase
N_2_O: nitrous oxide
OAC: ophthalmic artery chemotherapy
OCT: optical coherence tomography
RB: retinoblastoma
ET N_2_O: end tidal nitrous oxide
